# Operating room nurse's awareness and implementation status of the prevention of patient's intraoperative acquired pressure injuries: design and validation of a questionnaire

**DOI:** 10.3389/fsurg.2023.1308181

**Published:** 2024-01-04

**Authors:** Zhenya Zou, Shijiao Lv, Qian Gao, Xiaoyang Zhou, Jinbao Mao

**Affiliations:** ^1^ Operating Room, Shandong Provincial Hospital Affiliated to Shandong First Medical University, Jinan, China; ^2^ School of Nursing, Shandong First Medical University & Shandong Academy of Medical Sciences, Taian, China; ^3^ Specialty Care Outpatient, Shandong Provincial Hospital Affiliated to Shandong First Medical University, Jinan, China

**Keywords:** intraoperative acquired pressure injuries, operating room nursing, cognitive conditions, status of implementation, instrument development, surveys and questionnaires, validation study

## Abstract

**Aim:**

To compile the awareness and implementation status of patients with intraoperative acquired pressure injuries prevention by operating room nurses and to test its reliability and validity.

**Design:**

This is an equipment development research based on recommendations for developing a reliable and valid questionnaire.

**Methods:**

The research was carried out in two phases from February to November 2022. Through a panel discussion, expert consultation, and literature review, the questionnaire for operating room nurses on the current status of awareness and implementation of the prevention of intraoperative acquired pressure injuries was preliminarily formulated. The formal questionnaire was developed through validity analysis, reliability analysis and item analysis, and reliability and validity tests were conducted. Moreover, according to the questionnaire survey results, confirmatory factor analysis was carried out to construct the structural equation model.

**Results:**

The initial questionnaire consisted of five dimensions with 48 items, which was finalized to five dimensions with 38 items after reliability and validity testing and analysis. The five dimensions included implementation of intraoperative acquired pressure injuries prevention, intraoperative acquired pressure injuries preventing cognitive conditions, preoperative intraoperative acquired pressure injuries preventing cognitive conditions, basic knowledge of pressure injuries, and implementation of intraoperative acquired pressure injuries prevention in special patients. Cronbach's α of the overall questionnaire was 0.969 while that of each dimension was 0.846–0.959. The KMO value of structural validity was 0.945 (*P* < 0.001), and the contribution rate of cumulative variance was 70.694%. The fitting of confirmatory factor analysis was found to be generally ideal: *χ^2^*/*df *= 2.382, *RMR *= 0.027, *TLI *= 0.894, *RMSEA *= 0.072, *IFI *= 0.905, *CFI *= 0.904.

**Conclusions:**

The study and design of the questionnaire for operating room nurses on the current status of awareness and implementation of the prevention of intraoperative acquired pressure injuries are scientific and rational, providing a scientific basis for the standardized reform of hospitals and the optimization of the intraoperative acquired pressure injuries management system of the operating room.

## 1 Introduction

The prevention of intraoperative acquired pressure injuries (IAPI) is an important part of operating room care, which is also a global health issue of great concern. Several objective factors, such as the position during operation, the methods of anesthesia; and patient factors, such as individual tolerance capacity (1). It may cause a range of psychological problems and prolonged hospitalization (2, 3). In addition, it will increase the cost burden of patients and healthcare systems to some extent (4). Combine risk assessment prevention and prevention strategies to increase the nurses' awareness of pressure injuries (PI), thereby reducing the incidence of PI (5). This study intended to design a questionnaire for nurses in the operating room on the cognitive and operational status of IAPI, investigate the current status of IAPI prevention, and provide specific assessment tools for further optimizing prevention and management.

## 2 Background

### 2.1 Basic concepts and characteristics of pressure injury

IAPI often occurs within 48 to 72 h after operation, which is characterized by acupressure pale erythema, purple skin, and blistering (6). Factors such as intraoperative hypothermia and long surgical immobilization time increase the incidence of IAPI. Studies have shown that most patients with PI of varying degrees will occur after the operation time is greater than 4 h, and the risk of PI increases by 33% for every 0.5 h increase (7).

### 2.2 Staging of pressure injury

The first staging system was recognized by Shea in 1975. Subsequently, in 1991, the International Association of Enterostomal Therapists (IAET) simplified and refined the system of Shea (8), however, PI is divided into four stages as before: ruddy bruising, inflammatory infiltrates, superficial ulcers, and deep ulcers. In addition, the National Pressure Ulcer Advisory Panel (NPUAP) staging system is the most widely used staging scale for PI, which was last revised in 2016 (9). Depending on the current understanding of the etiology of PI and the recent release of ICD-11 by the World Health Organization (WHO) in 2018, PI is classified into six stages in NPUAP: nonblanchable erythema of intact skin, partial skin defects with the exposed dermis, full skin defects, full skin, and tissue defects, obscured full skin and tissue defects and persistent nonblanchable erythema of deep tissue (10).

### 2.3 Development of questionnaires on the prevention of pressure injuries

At present, the most widely used pressure injury risk assessment scales include the Braden scale, the Norton scale, and the Waterlow scale. Although these scales have a good effect on the application process, there are also some disadvantages. The Braden scale is currently the most widely used pressure injury risk assessment scale in the world, and although it is good for predicting pressure injury, it is not suitable for surgical patients (11). In contrast, the Norton scale and the Waterlow scale are more suitable for use in the operating room, and the Waterlow scale is more comprehensive and requires higher expertise from the evaluator (12). Moreover, the CORN-IAPI scale evaluates surgical patients from three aspects: preoperative, intraoperative and postoperative, including 2 dimensions and 10 factors, which are defined by anesthesia risk classification, body mass index (BMI), skin condition of the pressure site, preoperative limb activity, planned operation time, high-risk diseases, factors of body temperature loss, brought in PI, surgical blood loss, pressure shear force change, actual operation time, and postoperative skin results, and the scale is being promoted for use in China and has been shown to be effective in the prevention of IAPI (13).

With the improvement of measures to prevent IAPI, it is significant to discuss the understanding and implementation of operating room nurses on the prevention of IAPI in patients. A review of the literature revealed that most of the current studies have focused mainly on IAPI preventive measures or IAPI treatment strategies, ignoring the importance of the operating room nurse in the process of preventing or treating IAPI (14). Although some studies have investigated the awareness of IAPI among OR nurses, there is no specific research instrument to measure it (15). Scholars have developed IAPI-related test papers to determine OR nurses' IAPI knowledge through the scores of the test paper, which is very limited. Firstly, the content of the test papers produced by different scholars is not the same, and the test papers have not been developed through scientific and standardized methods, which lacks reliability and validity. On the other hand, there is a lack of specific tools to measure the current status of IAPI implementation among OR nurses.

## 3 The study

### 3.1 Aim

The aim of this study was to develop a questionnaire for operating room nurses on the current status of awareness and implementation of the prevention of IAPI. Moreover, it provides a reference for further improving the preventive measures of IAPI.

### 3.2 Study design

This was a methodological study of scales conducted in a multi-centre (China) between February and November 2022. The study was conducted in two phases, the first involving the design and commissioning of the questionnaire, and the second phase including a formal validation process.

### 3.3 Sample/participants

In both phases, the subjects involved a sample of 530 operating room nurses from three 3-A-class hospitals in Shandong Province, which are regional hospitals that can provide high-level specialized medical and health services and perform higher education and scientific research tasks. Inclusion criteria were as follows: ① have a nurse qualification certificate; ② voluntary participation in this study; ③ professional nursing in the operating room. Exclusion criteria were as follows: ① unable to attend on time due to further education or vacation.

The principle of determining the sample size of this study was as follows: the number of nurses included is 5–10 times the number of survey items and the sample number is ≥100 cases; The sample size taken by the structural equation model is at least 200 cases, and for each additional variable, the sample size increases by 5–10 times on the basis of the independent variable.

### 3.4 Procedure

#### 3.4.1 Preparation of questionnaires by operating room nurses on the awareness and implementation status of prevention of patient's acquired pressure injury during surgery

This study combined expert consultation method, literature analysis method and group discussion method to compile a questionnaire for operating room nurses on the cognition and implementation status of prevention of patient's acquired pressure injury. Through reviewing relevant literature at home and abroad, after intensive discussion by members of the research group, and combined with clinical post management measures, a questionnaire entry pool of 48 items in five dimensions including IAPI prevention implementation, IAPI preventive cognition, preoperative IAPI preventive cognition, basic knowledge of pressure injury, and special patient IAPI prevention implementation was preliminarily formed.

The expert consultation method was used to conduct 2 rounds of expert consultation for 10 experts, and the questionnaire on the cognition and implementation status of the nurses in the operating room on the prevention of patient's acquired PI was revised, and the expert inclusion criteria were as follows: ① engaged in clinical nursing or nursing management in the operating room; ② voluntary and guaranteed continuous participation in the subject; ③ have more than 10 years of clinical work experience; ④ intermediate or above professional title; ⑤ bachelor's degree or above. The experts were contacted before issuing the letter inquiry form through e-mail or on-site distribution to obtain their advice, and eventually, a questionnaire of prevention of IAPI containing 45 items in five dimensions for the cognition and implementation status of operating room nurses was developed.

#### 3.4.2 Reliability and validity test of the questionnaire of the operating room nurse on the prevention of patients' cognition and implementation status of intraoperative acquired pressure injury

The questionnaire of prevention of IAPI with five dimensions and 45 items to the cognition and implementation status of operating room nurses was verified through two stages. The first stage was as follows: operating room nurses from three 3-A-class hospitals in Shandong Province were selected to fill in the questionnaire for project analysis, reliability analysis and validity analysis. The questionnaire consisted of three parts: ① general profile of the study subject; ② purpose and description of the survey; ③ the main part of the questionnaire consists of 45 entries. A 5-point Likert scale was adopted for the 45 items. The higher the score, the clearer the understanding of the operating room nurse in the prevention of patient-acquired PI and the better able to implement it. Before the questionnaire is distributed, the research purpose and precautions were explained to the research objects. After the questionnaire was collected, the contents were carefully verified and incomplete questionnaires were eliminated. Based on the results of the initial questionnaire data analysis, the questionnaire was revised and entered the second stage. The second phase was as follows: operating room nurses from three 3-A-class general hospitals in Shandong Province were selected for the confirmatory factor analysis and structural equation construction. Nurses who had participated in the previous phase were excluded. Based on the research results, the questionnaire was revised, revealing a questionnaire of the prevention of patient's IAPI composed of five dimensions and 38 items that can be applied to the the operating room nurse's awareness and implementation status.

### 3.5 Statistical analysis

The data used are entered and proofread through Microsoft Excel. Demographic characteristics are described through descriptive statistics, such as the average of continuous variables and the frequency of category variables. Date analysis was performed using IBM SPSS software version 26.0, such as project analysis and reliability analysis, with a significance value of *p* set at <0.05. In addition, AMOS 24.0 statistical software was used to validate factor analysis and construct structural equation.

## 4 Results

### 4.1 General profile information about the study subject

The questionnaire was revised in two stages and 530 valid questionnaires were received. Table 1 shows general information about participants in both phases.

**Table 1:** Characteristics of the participants [phase 1 & phase 2; (*n* = 530)].

Item	*n*	%
Age		
≤25	104	19.6
26–30	128	24.2
31–35	166	31.3
36–40	90	17
>40	42	7.9
Gender		
Female	416	78.5
Male	114	21.5
Initial academic qualifications		
Secondary degree	72	13.6
Associate degree	124	23.4
Bachelor	326	61.5
Master's degree and above certification	8	1.5
Highest academic qualification		
Master's degree and above certification	26	4.9
Bachelor	490	92.5
Associate degree	10	1.9
Secondary degree	4	0.7
Professional title		
Nurse	114	21.5
Senior nurse	172	32.5
Supervisor nurse	232	43.8
Associate chief nurse	12	2.3
Level		
N0	2	0.4
N1	160	30.2
N2-1	94	17.7
N2-2	136	25.7
N3-1	102	19.2
N3-2	36	6.8
Whether or not a specialist team leader		
No	484	91.3
Yes	46	8.7
Specialist departments		
Gastroenterology	48	9.1
Hepatology	36	6.8
Joint surgery	20	3.8
Wound surgery	48	9.1
Hand and foot surgery	28	5.3
Spine surgery	16	3.0
Neurosurgery	34	6.4
Ophthalmology	22	4.2
Otorhinolaryngology	24	4.5
Oral surgery	14	2.6
Cardiac surgery	44	8.3
Thoracic surgery	36	6.8
Vascular surgery	26	4.9
Urinary surgery	40	7.5
Gynecology	40	7.5
Obstetric	12	2.3
Pediatrics	22	4.2
Liver transplantation	12	2.3
Robot	8	1.5
Post		
Surgical post	454	85.7
Logistics post	60	11.3
Management post	8	1.5
Other	8	1.5
Years of working experiences		
≤1	58	10.9
2–5	134	25.3
6–10	106	20.0
11–15	156	29.4
>15	76	14.3
Whether or not a subspecialty nurse		
No	402	75.8
Yes	128	24.2

### 4.2 Phase 1. Initial data analysis of the questionnaire

#### 4.2.1 Item analysis

Project analysis is used to assess the effectiveness and applicability of questionnaire items. The principle is to summarize the conditions first, with the first 27% of subjects recorded as high and the second 73% as low. The *T*-test is then used to compare the difference between high and low-score groups. If there is a difference, the design of the scale item is appropriate. Otherwise, the scale item is indistinguishable from the information and the design is unreasonable. It should be deleted (16).

As can be seen from Table 2, high and low scores showed significance for Q1 to Q45 items (*p* < 0.05), indicating that all 45 projects were well differentiated and did not require the deletion of the analysis items.

**Table 2:** The analysis results of the project analysis.

Title	Group mean score ± SD (%)		*T*-value	*P*-value
	Low score (*n* = 74)	High score (*n* = 72)		
Q1	3.4 ± 0.7	4.8 ± 0.5	−14.313	0.000*
Q2	3.3 ± 0.7	4.7 ± 0.5	−13.149	0.000*
Q3	3.2 ± 0.6	4.8 ± 0.5	−16.042	0.000*
Q4	3.2 ± 0.7	4.7 ± 0.6	−13.383	0.000*
Q5	3.8 ± 0.7	5.0 ± 0.1	−15.177	0.000*
Q6	3.6 ± 0.6	4.9 ± 0.4	−15.396	0.000*
Q7	4.0 ± 0.7	5.0 ± 0.0	−11.941	0.000*
Q8	3.8 ± 0.6	4.9 ± 0.2	−14.368	0.000*
Q9	4.0 ± 0.6	5.0 ± 0.1	−13.449	0.000*
Q10	3.7 ± 0.5	4.9 ± 0.3	−17.597	0.000*
Q11	3.7 ± 0.6	4.9 ± 0.3	−16.617	0.000*
Q12	3.8 ± 0.5	5.0 ± 0.1	−18.254	0.000*
Q13	3.5 ± 0.6	4.9 ± 0.3	−17.117	0.000*
Q14	3.8 ± 0.5	4.9 ± 0.3	−15.896	0.000*
Q15	3.6 ± 0.7	4.9 ± 0.3	−15.617	0.000*
Q16	4.1 ± 0.6	5.0 ± 0.0	−12.801	0.000*
Q17	4.0 ± 0.7	4.9 ± 0.4	−9.907	0.000*
Q18	4.0 ± 0.6	5.0 ± 0.1	−12.663	0.000*
Q19	3.7 ± 1.1	4.9 ± 0.4	−8.848	0.000*
Q20	4.1 ± 0.6	5.0 ± 0.1	−13.866	0.000*
Q21	3.8 ± 0.7	4.9 ± 0.3	−11.756	0.000*
Q22	4.0 ± 0.6	5.0 ± 0.1	−14.401	0.000*
Q23	3.7 ± 0.8	4.8 ± 0.5	−10.528	0.000*
Q24	4.0 ± 0.6	5.0 ± 0.0	−14.414	0.000*
Q25	3.9 ± 0.6	4.9 ± 0.4	−11.698	0.000*
Q26	3.9 ± 0.5	5.0 ± 0.0	−11.698	0.000*
Q27	3.7 ± 0.7	4.9 ± 0.3	−13.619	0.000*
Q28	3.8 ± 0.6	5.0 ± 0.0	−17.381	0.000*
Q29	3.6 ± 0.7	4.9 ± 0.2	−15.340	0.000*
Q30	3.9 ± 0.5	5.0 ± 0.0	−17.614	0.000*
Q31	3.9 ± 0.5	5.0 ± 0.2	−15.893	0.000*
Q32	3.9 ± 0.5	5.0 ± 0.1	−19.053	0.000*
Q33	3.9 ± 0.7	4.9 ± 0.4	−11.258	0.000*
Q34	3.9 ± 0.5	5.0 ± 0.1	−17.078	0.000*
Q35	3.9 ± 0.7	4.9 ± 0.2	−13.053	0.000*
Q36	4.0 ± 0.5	5.0 ± 0.2	−16.100	0.000*
Q37	3.8 ± 0.7	4.9 ± 0.3	−13.934	0.000*
Q38	3.9 ± 0.5	5.0 ± 0.2	−18.694	0.000*
Q39	3.7 ± 0.7	4.8 ± 0.6	−9.841	0.000*
Q40	4.1 ± 0.5	5.0 ± 0.0	−14.561	0.000*
Q41	4.0 ± 0.7	5.0 ± 0.2	−10.440	0.000*
Q42	4.1 ± 0.5	5.0 ± 0.2	−15.041	0.000*
Q43	4.0 ± 0.5	4.9 ± 0.3	−11.654	0.000*
Q44	4.1 ± 0.5	5.0 ± 0.0	−14.720	0.000*
Q45	4.2 ± 0.5	5.0 ± 0.2	−11.689	0.000*

* Indicated significance set at.01.

#### 4.2.2 Reliability analysis

Reliability analysis, which is primarily used to evaluate the reliability and accuracy of quantitative data answers, should be guided by the following principles: (1) the Cronbach *α* coefficient ≥0.8, indicating high reliability; (2) if Corrected Item-Total Correlation (CITC) ≤0.3, consider deleting the item; and (3) if the “deleted *α* coefficient” is significantly higher than the *α* coefficient, consider deleting the item and re-analyzing it. Details are shown in Table 3.

**Table 3:** Reliability analysis.

Title	Corrected item-total correlation (CITC)	Cronbach's alpha if item deleted	Title	Corrected item-total correlation (CITC)	Cronbach's alpha if item deleted	Cronbach's α
Q1	0.544	0.970	Q24	0.758	0.969	0.970
Q2	0.592	0.970	Q25	0.585	0.970	
Q3	0.605	0.970	Q26	0.769	0.969	
Q4	0.579	0.970	Q27	0.587	0.970	
Q5	0.665	0.970	Q28	0.792	0.969	
Q6	0.650	0.970	Q29	0.690	0.970	
Q7	0.651	0.970	Q30	0.750	0.969	
Q8	0.628	0.970	Q31	0.679	0.970	
Q9	0.688	0.970	Q32	0.770	0.969	
Q10	0.692	0.970	Q33	0.624	0.970	
Q11	0.666	0.970	Q34	0.765	0.969	
Q12	0.728	0.969	Q35	0.663	0.970	
Q13	0.708	0.969	Q36	0.741	0.969	
Q14	0.723	0.969	Q37	0.685	0.970	
Q15	0.707	0.969	Q38	0.766	0.969	
Q16	0.656	0.970	Q39	0.585	0.970	
Q17	0.530	0.970	Q40	0.735	0.970	
Q18	0.683	0.970	Q41	0.632	0.970	
Q19	0.483	0.971	Q42	0.562	0.970	
Q20	0.702	0.970	Q43	0.624	0.970	
Q21	0.556	0.970	Q44	0.594	0.970	
Q22	0.676	0.970	Q45	0.590	0.970	
Q23	0.474	0.971				

Standardized Cronbach's Alpha:0.973.

As can be seen from Table 3, the reliability coefficient value is 0.970, as it is greater than 0.9, indicating a very high reliability quality of the study data. For the “deleted item alpha factor”, the reliability coefficient would increase significantly if Q19 and Q23 were deleted, so consider correcting or deleting them. For the “CITC value”, all items meet the standard.

In summary, consider deleting Q19 and Q23.

#### 4.2.3 Validity analysis

Validity analysis is used to determine the relevance of tool items to the concepts being assessed (17). Content validity and structural validity are selected for analysis in this study.

##### 4.2.3.1 Content validity

The average content validity index (S-CVI) of the questionnaire was 0.926, and the content validity index (I-CVI) of the entry level was 0.821∼1.000.

##### 4.2.3.2 Structural validity

The validity analysis of the data was verified by a comprehensive analysis of KMO, commonality, variance explanation rate, and factor loading coefficient.

The KMO test and the Bartlett test allow for assessing the applicability factor analysis for a particular data or the adequacy of sampling (18). Common values are used to eliminate irrational research projects; variance explanation rates are used to illustrate the level of information extraction; and the factor loading coefficients are used to measure the correlation between factors and problems.

The KMO value is 0.946 with a result greater than 0.8, indicating good validity of the study data. As can be seen from Table 4, two factors in Q8, Q10, Q42, and Q44 load simultaneously >0.5, while factors in Q17 load less than 0.5, so the five items with invalid headings should be deleted. In addition, the variance interpretation rate values of the six factors were 18.29%, 14.88%, 13.95%, 12.51%, 6.21%, and 5.26%, respectively. The cumulative variance explanation rate after rotation is 71.10% > 60%. This means that the amount of information on the research item can be extracted efficiently.

**Table 4:** Results of validity analysis.

Title	Factor loading (Rotated)						Communality
	Factor 1	Factor 2	Factor 3	Factor 4	Factor 5	Factor 6	
Q1	0.215	0.007	0.138	0.814	0.121	0.033	0.743
Q2	0.202	0.188	0.108	0.767	0.043	0.110	0.689
Q3	0.134	0.204	0.207	0.799	0.018	0.073	0.746
Q4	0.122	0.178	0.180	0.785	0.073	0.061	0.704
Q5	0.418	0.057	0.564	0.364	0.022	0.114	0.642
Q6	0.087	0.141	0.441	0.674	0.120	0.171	0.720
Q7	0.350	0.062	0.793	0.178	0.061	0.070	0.796
Q8	0.152	0.147	0.564	0.525	0.012	0.086	0.646
Q9	0.391	0.073	0.785	0.155	0.119	0.106	0.824
Q10	0.190	0.200	0.592	0.516	0.082	0.046	0.702
Q11	0.143	0.185	0.629	0.492	0.032	0.112	0.705
Q12	0.425	0.165	0.685	0.281	0.018	0.076	0.762
Q13	0.094	0.302	0.465	0.614	0.079	0.186	0.734
Q14	0.336	0.236	0.666	0.268	0.091	0.085	0.700
Q15	0.115	0.309	0.492	0.595	0.066	0.120	0.723
Q16	0.383	0.062	0.762	0.145	0.064	0.139	0.776
Q17	0.017	0.307	0.395	0.174	0.377	0.214	0.468
Q18	0.651	0.059	0.380	0.172	0.318	0.059	0.705
Q19	0.183	0.227	0.050	0.077	0.740	0.268	0.713
Q20	0.713	0.151	0.325	0.086	0.322	0.076	0.753
Q21	0.176	0.345	0.138	0.089	0.789	0.091	0.807
Q22	0.685	0.254	0.176	0.092	0.380	0.027	0.717
Q23	0.221	0.353	−0.001	0.089	0.751	−0.051	0.748
Q24	0.744	0.253	0.269	0.172	0.178	0.135	0.770
Q25	0.200	0.679	0.022	0.168	0.313	0.051	0.631
Q26	0.726	0.318	0.241	0.277	0.075	0.049	0.771
Q27	0.233	0.671	−0.046	0.307	0.139	0.057	0.623
Q28	0.688	0.418	0.235	0.241	0.068	0.110	0.778
Q29	0.266	0.737	0.087	0.279	0.140	0.080	0.725
Q30	0.678	0.422	0.288	0.130	0.034	0.094	0.748
Q31	0.348	0.647	0.180	0.089	0.242	0.099	0.649
Q32	0.680	0.420	0.280	0.133	−0.010	0.235	0.790
Q33	0.273	0.663	0.235	0.086	0.042	0.135	0.596
Q34	0.693	0.461	0.225	0.099	0.064	0.186	0.792
Q35	0.280	0.696	0.191	0.028	0.268	0.155	0.696
Q36	0.697	0.355	0.231	0.140	0.085	0.183	0.725
Q37	0.327	0.704	0.082	0.219	0.154	0.102	0.691
Q38	0.701	0.410	0.275	0.154	0.100	0.043	0.770
Q39	0.185	0.676	0.134	0.131	0.167	0.110	0.566
Q40	0.649	0.302	0.287	0.153	0.008	0.334	0.730
Q41	0.198	0.491	0.236	0.149	0.088	0.528	0.644
Q42	0.529	0.058	0.067	0.189	0.154	0.563	0.665
Q43	0.185	0.496	0.142	0.159	0.196	0.555	0.672
Q44	0.547	0.055	0.118	0.202	0.063	0.643	0.775
Q45	0.094	0.413	0.279	0.141	0.158	0.600	0.662
Eigen value (Unrotated)	20.890	4.058	2.814	1.591	1.377	1.265	-
% of Variance (Unrotated)	46.42%	9.02%	6.25%	3.54%	3.06%	2.81%	-
Cumulative % of Variance (Unrotated)	46.42%	55.44%	61.70%	65.23%	68.29%	71.10%	-
Eigen value (Rotated)	8.229	6.695	6.279	5.631	2.794	2.366	-
% of Variance (Rotated)	18.29%	14.88%	13.95%	12.51%	6.21%	5.26%	-
Cumulative % of Variance (Rotated)	18.29%	33.17%	47.12%	59.63%	65.84%	71.10%	-
KMO	0.946						-
Bartlett's Test of Sphericity	11,295.984						-
df	990						-
*P* value	0						-

The blue numbers in the table indicate that the absolute value of the factor loading is greater than 0.5.

In summary, seven questions were excluded from the analysis of the initial questionnaire: Q8, Q10, Q17, Q19, Q23, Q42, and Q44. At the same time, the remaining questions were adjusted and the questionnaire was sent out again for hypothesis verification.

### 4.3 Phase 2. Formal validation and final questionnaire

#### 4.3.1 Reliability analysis

After the reliability analysis of the revised questionnaire, the overall Cronbach's α coefficient was 0.969. Moreover, Cronbach's alpha coefficients for each dimension were as follows: implementation of IAPI prevention, 0.927; IAPI prevents cognitive conditions, 0.959; preoperative IAPI to prevent cognitive conditions, 0.939; basic knowledge of PI, 0.926; implementation of IAPI prevention in special patients, 0.846. All indicators were above 0.7, indicating good reliability value of the questionnaire.

#### 4.3.2 Factor analysis

Principal component analysis was performed on the revised questionnaire data to test the validity of the variables. Before factor analysis, each variable was tested for the KMO test and Bartlett's test of sphericity to determine if factor analysis is possible.

The KMO value is 0.945 > 0.6, and the significance value of the Bartlett sphericity test is <0.05, indicating that there are common factors and are suitable for factor analysis.

A total of 5 factors were extracted using principal component analysis, with a cumulative variance interpreted as 0.71, indicating that of all variables, 71% of variable information could be aggregated by extracting 5 factors, as detailed in Table 5. By factor rotation, the maximum variance method is used and the factor load of each component was greater than 0.5, as shown in Table 6. A total of five components were identified, which is in line with the revised questionnaire. In conclusion, the structure of this survey questionnaire is reasonable.

**Table 5:** Total variance explained.

Component	Initial eigenvalues			Extraction sums of squared loadings			Rotation sums of squared loadings		
	Total	% of variance	Cumulative %	Total	% of variance	Cumulative %	Total	% of variance	Cumulative %
1	17.897	47.096	47.096	17.897	47.096	47.096	6.911	18.186	18.186
2	3.644	9.589	56.685	3.644	9.589	56.685	6.785	17.855	36.041
3	2.619	6.892	63.577	2.619	6.892	63.577	5.550	14.606	50.646
4	1.466	3.857	67.434	1.466	3.857	67.434	4.982	13.111	63.757
5	1.239	3.261	70.694	1.239	3.261	70.694	2.636	6.937	70.694
6	0.964	2.538	73.232						
7	0.795	2.093	75.325						
8	0.780	2.053	77.378						
9	0.677	1.781	79.159						
10	0.605	1.593	80.752						
11	0.553	1.456	82.207						
12	0.520	1.369	83.577						
13	0.498	1.311	84.887						
14	0.471	1.240	86.127						
15	0.438	1.154	87.281						
16	0.392	1.031	88.312						
17	0.372	0.980	89.292						
18	0.321	0.845	90.137						
19	0.312	0.821	90.958						
20	0.283	0.746	91.704						
21	0.279	0.735	92.439						
22	0.267	0.702	93.141						
23	0.251	0.660	93.800						
24	0.244	0.642	94.442						
25	0.229	0.603	95.045						
26	0.210	0.553	95.598						
27	0.203	0.534	96.132						
28	0.189	0.497	96.629						
29	0.177	0.467	97.096						
30	0.166	0.437	97.533						
31	0.151	0.397	97.930						
32	0.145	0.381	98.311						
33	0.139	0.366	98.677						
34	0.122	0.322	98.999						
35	0.116	0.305	99.304						
36	0.102	0.268	99.572						
37	0.093	0.244	99.816						
38	0.070	0.184	100.000						

Extraction method: principal component analysis.

**Table 6:** Rotated component matrix^a^.

	Component				
	1	2	3	4	5
B21	0.648				
B22	0.635				
B24	0.702				
B26	0.624				
B28	0.618				
B30	0.687				
B32	0.663				
B34	0.662				
B36	0.646				
B37	0.675				
B38	0.633				
B15		0.646			
B17		0.700			
B20		0.725			
B23		0.681			
B25		0.686			
B27		0.687			
B29		0.715			
B31		0.718			
B33		0.730			
B35		0.651			
B5			0.572		
B7			0.770		
B8			0.780		
B9			0.607		
B10			0.689		
B12			0.675		
B14			0.773		
B1				0.824	
B2				0.779	
B3				0.813	
B4				0.794	
B6				0.659	
B11				0.596	
B13				0.589	
B16					0.753
B18					0.779
B19					0.778

Extraction Method: Principal Component Analysis.

Rotation Method: Varimax with Kaiser Normalization.

^a^ Rotation converged in 9 iterations.

#### 4.3.3 Confirmatory factor analysis

**Figure 1:**
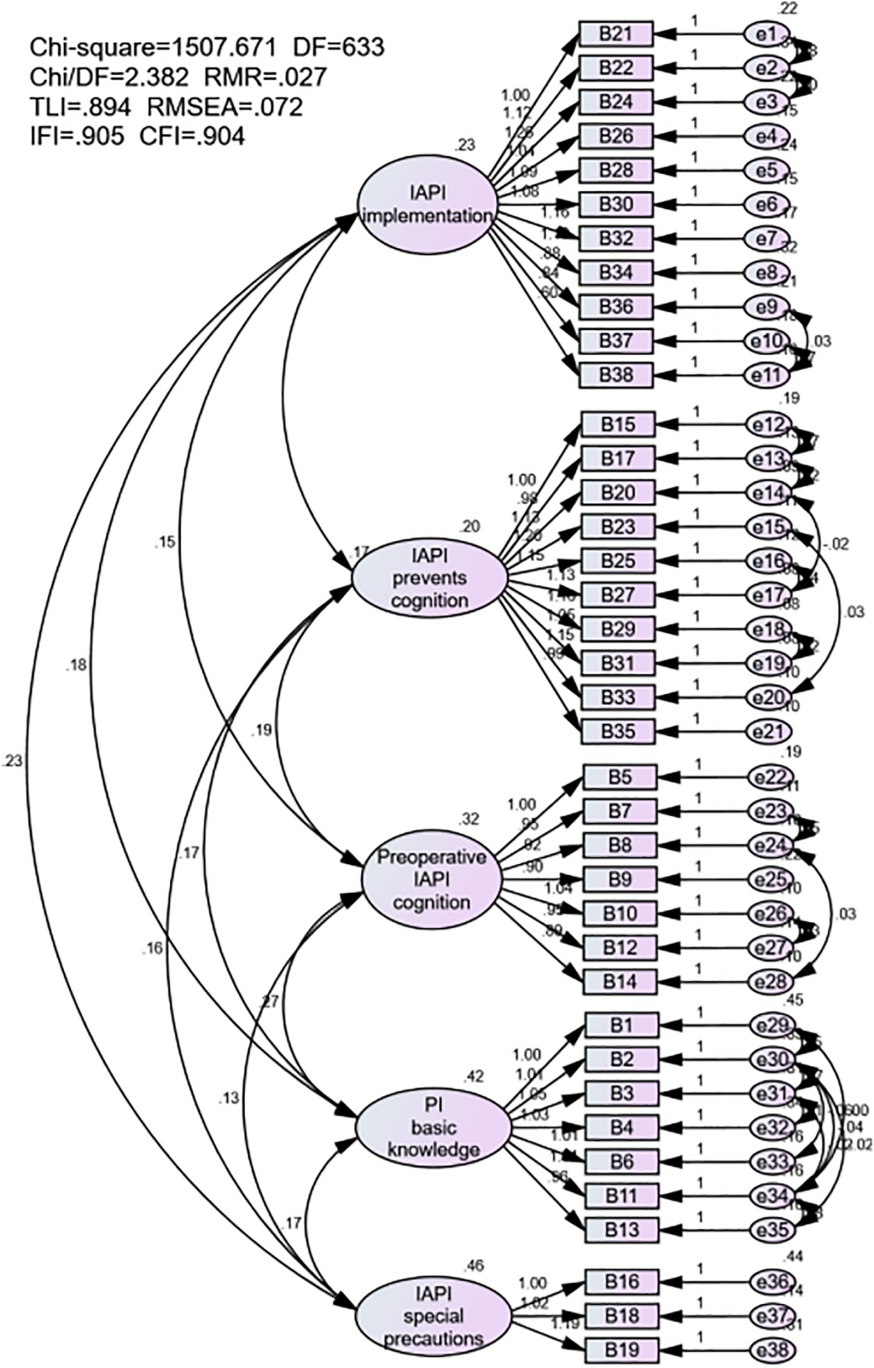
Model of structural equations.

In this study, we performed a validation factor analysis using AMOS 24.0 software for a questionnaire on the patients’ cognition and implementation status of surgical acquired pressure injury among operating room nurses. Key indications included: Root Mean Square Residual (RMR), Tucker-Lewis Index (TLI), Incremental Fit Index (IFI), Comparative Fit Index (CFI), and Root-Mean-Square Error of Approximation (RMSEA). By testing the goodness-of-fit coefficient of the model, the results show that all indicators are within a reasonable range. The model path is significantly tested, the model factor load is greater than 0.5, and the path is significant, which proves once again that the model has good structural validity. The AMOS verification model is shown in Figure 1, and the path fit and path coefficient are shown in Tables 7, 8.

**Table 7:** Model fit summary.

Model	Critical value	Data for test results	Judgment of model adaptation
χ^2^/df	<3.00	2.382	Yes
RMR	<0.05	0.027	Yes
TLI	>0.80	0.894	Yes
IFI	>0.90	0.905	Yes
CFI	>0.90	0.904	Yes
RMSEA	<0.08	0.072	Yes

**Table 8:** Path coefficient analysis.

Path relationships			Estimate	AVE	CR
B21: Do you think it is important to observe the color and swelling of the skin in the area where the patient is compressed?	<—	IAPI implementation	0.720	0.532	0.926
B22: Do you decompress the patient's area of pressure at least every 2 h during the procedure, without medical contraindications and with the consent of the surgeon?	<—	IAPI implementation	0.681		
B24: Do you decompress your patient's skin by moving or adjusting palpable non-surgical compression areas, positional pads, etc. during surgery?	<—	IAPI implementation	0.792		
B26: Do you use the appropriate type, material, and model of instruments during the procedure according to the patient's body shape and local skin condition?	<—	IAPI implementation	0.788		
B28: Do you give patients the right to wear and immobilize instruments in surgical patient care?	<—	IAPI implementation	0.736		
B30: Do you apply prophylactic dressings or pads for protection before using the device?	<—	IAPI implementation	0.804		
B32: Do you regularly monitor the tightness of your medical devices during surgery?	<—	IAPI implementation	0.809		
B34: Do you move or adjust your instruments in small areas at least every 2 h during surgery?	<—	IAPI implementation	0.710		
B36: Do you think it is important to conduct an intraoperative risk assessment of the patient according to the CORN-IAPI assessment scale, with relevant preventive measures based on the patient's risk level?	<—	IAPI implementation	0.679		
B37: Do you taking steps to prevent intraoperative hypothermia in your surgical patient care?	<—	IAPI implementation	0.686		
B38: Do you check the condition of the skin in the area where the patient is compressed, and accurately record and hand it over after the procedure?	<—	IAPI implementation	0.588		
B15: Do you think prophylactic dressings are important for patients at risk of IAPI with extreme obesity (BMI > 40), or surgery time > 6 h, or age > 75 years?	<—	IAPI prevents cognition	0.722	0.694	0.958
B17: Do you think it is important to choose and use prophylactic dressings, decompression pads, etc. for skin decompression in patients at high risk of IAPI?	<—	IAPI prevents cognition	0.775		
B20: Do you think it is important to observe the color and swelling of the skin in the area where the patient is compressed?	<—	IAPI prevents cognition	0.861		
B23: Do you think it is important to decompress the patient's skin by moving or adjusting the palpable non-surgical compression area, position pad, etc. during surgery?	<—	IAPI prevents cognition	0.857		
B25: Do you think it is important to select the appropriate type, material, and model of instruments to prevent device-related pressure injuries in surgical patient care based on the patient's body shape and local skin condition?	<—	IAPI prevents cognition	0.831		
B27: Do you think it is important to properly wear and immobilize the device to prevent device-related pressure injuries in patients?	<—	IAPI prevents cognition	0.872		
B29: Do you think it is important to protect against device-related pressure injuries with a prophylactic dressing or pad before using the device?	<—	IAPI prevents cognition	0.874		
B31: Do you think it is important to regularly monitor the tightness of medical devices during surgery to prevent device-related pressure injuries?	<—	IAPI prevents cognition	0.857		
B33: Do you think it is important to move or adjust the instrument in small areas at least every 2 h without affecting the surgery to prevent device-related pressure injuries?	<—	IAPI prevents cognition	0.850		
B35: Do you think preventing maceration of the patient's skin is important to prevent the patient's acquired pressure injury during surgery?	<—	IAPI prevents cognition	0.820		
B5: Do you think it is important to know the patient's general profile, such as age, body mass index (BMI), physical activity, current risk level of pressure injury, previous or existing pressure injury, diabetes, history of cardiovascular and cerebrovascular diseases, etc.?	<—	Preoperative IAPI cognition	0.790	0.679	0.937
B7: Do you think it is important to know the patient's surgical situation, such as the type of surgery, estimated length of surgery, surgical position, anesthesia method, etc. before surgery?	<—	Preoperative IAPI cognition	0.845		
B8: Do you think it is important to evaluate the color, temperature, integrity, presence of edema, tenderness, etc. of the patient's whole body before surgery, and focus on the skin of the compressed area related to the surgical position?	<—	Preoperative IAPI cognition	0.858		
B9: Do you focus on understanding the condition of the patient's skin at the site of compression in relation to the surgical position before surgery?	<—	Preoperative IAPI cognition	0.731		
B10: Do you think it is important to conduct a preoperative risk assessment of the patient according to the CORN-IAPI assessment scale, with relevant preventive measures based on the patient's risk level?	<—	Preoperative IAPI cognition	0.877		
B12: Do you think it is important to apply a dressing under the disinfected area before surgery and remove the dressing after disinfection to prevent maceration of the patient's skin and prevent IAPI?	<—	Preoperative IAPI cognition	0.815		
B14: Do you think it is important to use the pressure relief tool correctly, choose and use positional cushions such as headrests, knee pillows, shoulder pads, chest pads, and heel pads to disperse the skin pressure of surgical patients?	<—	Preoperative IAPI cognition	0.843		
B1: Can you accurately distinguish between intraoperative acquired pressure injuries, inductive pressure injuries, and device-related pressure injuries?	<—	PI basic knowledge	0.694	0.607	0.915
B2: Can you accurately identify the stage of a surgical acquired pressure injury?	<—	PI basic knowledge	0.753		
B3: Are you proficient in timing the assessment of the risk of acquired pressure injury?	<—	PI basic knowledge	0.774		
B4: Are you proficient in using the CORN Acquired Stress Injury Risk Assessment Scale?	<—	PI basic knowledge	0.753		
B6: Are you proficient in the general profile of the surgical patient before surgery, such as age, body mass index (BMI), limb activity, existing pressure injury risk level, previous or existing pressure injury, diabetes, history of cardiovascular and cerebrovascular diseases, etc.?	<—	PI basic knowledge	0.853		
B11: Are you proficient in the preoperative risk assessment level of your patients before surgery and taking appropriate precautions according to the patient's risk level?	<—	PI basic knowledge	0.852		
B13: Are you proficient in determining the patient's intraoperative risk assessment level and taking appropriate preventive measures according to the patient's risk level?	<—	PI basic knowledge	0.839		
B16: Do you have prophylactic dressings for skin protection in surgical patient care for patients at intermediate risk of IAPI with extreme obesity (BMI > 40), or surgery time > 6 h, or age > 75 years?	<—	IAPI special precautions	0.717	0.656	0.850
B18: Do you use prophylactic dressings, decompression pads, etc. for skin decompression for patients at high risk of IAPI in your surgical patient care?	<—	IAPI special precautions	0.881		
B19: Do you use prophylactic dressings for skin protection in diabetic surgery patients in surgical patient care?	<—	IAPI special precautions	0.823		

The data after the correction of the model shows that: *χ^2^*/*df *= 2.382; RMR = 0.027; TLI = 0.894; RMSEA = 0.072; IFI = 0.905; CFI = 0.904. The overall display model structure is well valid, as shown in Figure 1. The final version of the questionnaire is shown in Table 9.

**Table 9:** The final version of the questionnaire.

Questionnaire on the status of awareness and implementation of the prevention of intraoperative acquired pressure injuries in patients by operating room nurses
1. Can you accurately distinguish between intraoperative acquired pressure injuries, inductive pressure injuries, and device-related pressure injuries?
① Very uncertain ② Uncertain ③ Rather certain ④ Certain ⑤ Very certain
2. Can you accurately identify the stage of a surgical acquired pressure injury?
① Very uncertain ② Uncertain ③ Rather certain ④ Certain ⑤ Very certain
3. Are you proficient in timing the assessment of the risk of acquired pressure injury?
① Very unskilled ② Incompetent ③ Relatively skilled ④ Skilled ⑤ Very skilled
4. Are you proficient in using the CORN Acquired Stress Injury Risk Assessment Scale?
① Very unskilled ② Incompetent ③ Relatively skilled ④ Skilled ⑤ Very skilled
5. Do you think it is important to know the patient's general profile, such as age, body mass index (BMI), physical activity, current risk level of pressure injury, previous or existing pressure injury, diabetes, history of cardiovascular and cerebrovascular diseases, etc.?
①Very unimportant ② Unimportant ③ Relatively important ④ Important ⑤ Very important
6. Are you proficient in the general profile of the surgical patient before surgery, such as age, body mass index (BMI), limb activity, existing pressure injury risk level, previous or existing pressure injury, diabetes, history of cardiovascular and cerebrovascular diseases, etc.?
① Very unskilled ② Incompetent ③ Relatively skilled ④ Skilled ⑤ Very skilled
7. Do you think it is important to know the patient's surgical situation, such as the type of surgery, estimated length of surgery, surgical position, anesthesia method, etc. before surgery?
① Very unimportant ② Unimportant ③ Relatively important ④ Important ⑤ Very important
8. Do you think it is important to evaluate the color, temperature, integrity, presence of edema, tenderness, etc. of the patient's whole body before surgery, and focus on the skin of the compressed area related to the surgical position?
① Very unimportant ② Unimportant ③ Relatively important ④ Important ⑤ Very important
9. Do you focus on understanding the condition of the patient's skin at the site of compression in relation to the surgical position before surgery?
① Very little knowledge ② No knowledge ③ Some knowledge ④ Knowledge ⑤ Very much knowledge
10. Do you think it is important to conduct a preoperative risk assessment of the patient according to the CORN-IAPI assessment scale, with relevant preventive measures based on the patient's risk level?
① Very unimportant ② Unimportant ③ Relatively important ④ Important ⑤ Very important
11. Are you proficient in the preoperative risk assessment level of your patients before surgery and taking appropriate precautions according to the patient's risk level?
① Very unskilled ② Incompetent ③ Relatively skilled ④ Skilled ⑤ Very skilled
12. Do you think it is important to apply a dressing under the disinfected area before surgery and remove the dressing after disinfection to prevent maceration of the patient's skin and prevent IAPI?
① Very unimportant ② Unimportant ③ Relatively important ④ Important ⑤ Very important
13. Are you proficient in determining the patient's intraoperative risk assessment level and taking appropriate preventive measures according to the patient's risk level?
① Very unskilled ② Incompetent ③ Relatively skilled ④ Skilled ⑤ Very skilled
14. Do you think it is important to use the pressure relief tool correctly, choose and use positional cushions such as headrests, knee pillows, shoulder pads, chest pads, and heel pads to disperse the skin pressure of surgical patients?
① Very unimportant ② Unimportant ③ Relatively important ④ Important ⑤ Very important
15. Do you think prophylactic dressings are important for patients at risk of IAPI with extreme obesity (BMI > 40), or surgery time > 6 h, or age > 75 years?
① Very unimportant ② Unimportant ③ Relatively important ④ Important ⑤ Very important
16. Do you have prophylactic dressings for skin protection in surgical patient care for patients at intermediate risk of IAPI with extreme obesity (BMI > 40), or surgery time > 6 h, or age > 75 years?
① Nearly always ② Sometimes ③ Sometimes ④ Most of the time ⑤ All of the time
17. Do you think it is important to choose and use prophylactic dressings, decompression pads, etc. for skin decompression in patients at high risk of IAPI?
① Very unimportant ② Unimportant ③ Relatively important ④ Important ⑤ Very important
18. Do you use prophylactic dressings, decompression pads, etc. for skin decompression for patients at high risk of IAPI in your surgical patient care?
① Nearly always ② Sometimes ③ Sometimes ④ Most of the time ⑤ All of the time
19. Do you use prophylactic dressings for skin protection in diabetic surgery patients in surgical patient care?
① Nearly always ② Sometimes ③ Sometimes ④ Most of the time ⑤ All of the time
20. Do you think it is important to observe the color and swelling of the skin in the area where the patient is compressed?
① Very unimportant ② Unimportant ③ Relatively important ④ Important ⑤ Very important
21. Do you regularly observe the colour and swelling of the patient's pressure area during the procedure?
① Nearly always ② Sometimes ③ Sometimes ④ Most of the time ⑤ All of the time
22. Do you decompress the patient's area of pressure at least every 2 h during the procedure, without medical contraindications and with the consent of the surgeon?
① Nearly always ② Sometimes ③Sometimes ④ Most of the time ⑤ All of the time
23. Do you think it is important to decompress the patient's skin by moving or adjusting the palpable non-surgical compression area, position pad, etc. during surgery?
① Very unimportant ② Unimportant ③ Relatively important ④ Important ⑤ Very important
24. Do you decompress your patient's skin by moving or adjusting palpable non-surgical compression areas, positional pads, etc. during surgery?
① Nearly always ② Sometimes ③ Sometimes ④ Most of the time ⑤ All of the time
25. Do you think it is important to select the appropriate type, material, and model of instruments to prevent device-related pressure injuries in surgical patient care based on the patient's body shape and local skin condition?
① Very unimportant ② Unimportant ③ Relatively important ④Important ⑤Very important
26. Do you use the appropriate type, material, and model of instruments during the procedure according to the patient's body shape and local skin condition?
① Nearly always ② Sometimes ③ Sometimes ④ Most of the time ⑤ All of the time
27. Do you think it is important to properly wear and immobilize the device to prevent device-related pressure injuries in patients?
① Very unimportant ② Unimportant ③ Relatively important ④ Important ⑤ Very important
28.Do you give patients the right to wear and immobilize instruments in surgical patient care?
① Nearly always ② Sometimes ③ Sometimes ④ Most of the time ⑤ All of the time
29. Do you think it is important to protect against device-related pressure injuries with a prophylactic dressing or pad before using the device?
①Very unimportant ② Unimportant ③ Relatively important ④ Important ⑤ Very important
30. Do you apply prophylactic dressings or pads for protection before using the device?
① Nearly always ② Sometimes ③ Sometimes ④ Most of the time ⑤ All of the time
31. Do you think it is important to regularly monitor the tightness of medical devices during surgery to prevent device-related pressure injuries?
① Very unimportant ② Unimportant ③ Relatively important ④ Important ⑤ Very important
32. Do you regularly monitor the tightness of your medical devices during surgery?
① Nearly always ② Sometimes ③ Sometimes ④ Most of the time ⑤ All of the time
33. Do you think it is important to move or adjust the instrument in small areas at least every 2 h without affecting the surgery to prevent device-related pressure injuries?
① Very unimportant ② Unimportant ③ Relatively important ④ Important ⑤ Very important
34. Do you move or adjust your instruments in small areas at least every 2 h during surgery?
① Nearly always ② Sometimes ③ Sometimes ④ Most of the time ⑤ All of the time
35. Do you think preventing maceration of the patient's skin is important to prevent the patient's acquired pressure injury during surgery?
① Very unimportant ② Unimportant ③ Relatively important ④ Important ⑤ Very important
36. Do you think it is important to conduct an intraoperative risk assessment of the patient according to the CORN-IAPI assessment scale, with relevant preventive measures based on the patient's risk level?
① Very unimportant ② Unimportant ③ Relatively important ④ Important ⑤ Very important
37. Do you taking steps to prevent intraoperative hypothermia in your surgical patient care?
① Nearly always ② Sometimes ③ Sometimes ④ Most of the time ⑤ All of the time
38. Do you check the condition of the skin in the area where the patient is compressed, and accurately record and hand it over after the procedure?
① Nearly always ② Sometimes ③ Sometimes ④ Most of the time ⑤ All of the time

## 5 Discussion

### 5.1 The significant nature of the questionnaire of operating room nurses on the cognition and implementation status of the prevention of patient IAPI

The operating room is an important department that is independently managed in the operation of the hospital, and the patient's time in the operating room is usually an independent event during the hospitalization, and IAPI mostly occurs within hours to up to 5 days after surgery (19). Continuously refining the nursing management mode of the operating room and improving the prevention awareness of the nursing staff in the operating room is the key to strengthening standardized nursing.

At present, although there are relevant scales to assess IAPI, they are less involved in the cognition of prevention related to operating room nurses, and a systematic prevention management questionnaire has not been formed. This study conducted in-depth research on the cognition and implementation status of IAPI prevention in patients in operating room nurses, analyzed various influencing factors of IAPI prevention, understood the implementation status of preventive measures, and provided the reference for further optimizing the prevention and management of IAPI through scientific evaluation.

### 5.2 The scientific nature of the questionnaire of operating room nurses on the cognition and implementation status of the prevention of patient’s IAPI

The overall Cronbach's alpha coefficient of the questionnaire was 0.969, whereas that of each dimension of the questionnaire was above 0.70, which is consistent with the criterion that the reliability coefficient should preferably be above 0.70. This indicated that the internal consistency of the questionnaire is great.

In this study, the questionnaire for operating room nurses on the current status of awareness and implementation of the prevention of IAPI was constructed using the expert consultation method, literature analysis method, and group discussion method. The scientific and reasonable verification of the entries through structural validity, exploratory factor analysis, and validation factor analysis confirmed that each dimension and item met the research theme. In the exploratory factor analysis, the principal component analysis method was used, and five principal components were obtained, which cumulatively explained 70.694% of the total variance. The factor load matrix of each factor is 0.572∼0.824, which met the requirements that the factor load should be greater than 0.400. Additionally, validation factor analysis showed that *χ^2^*/*df *= 2.382, RMR = 0.027, TLI = 0.894, RMSEA = 0.072, IFI = 0.905, CFI = 0.904 met the statistical criteria, which indicates that this model fits well and has a better simulation degree. Thus, the questionnaire structure has a great fit, and the structural validity of the questionnaire is excellent.

## 6 Conclusions

The questionnaire for operating room nurses on the current status of awareness and implementation of the prevention of IAPI is beneficial for nursing managers to understand the current mastery of IAPI prevention knowledge and the implementation of nursing measures by operating room nurses, and to investigate the areas in which IAPI preventive management can be improved in operating room nursing management. Thereby, this study provides a reference basis for the optimization of scientific preventive management in the operating room.

## Data Availability

The original contributions presented in the study are included in the article/Supplementary Material, further inquiries can be directed to the corresponding author.
